# Apoptotic cells in mouse blastocysts are eliminated by neighbouring blastomeres

**DOI:** 10.1038/s41598-021-88752-0

**Published:** 2021-04-29

**Authors:** Jozef Pisko, Alexandra Špirková, Štefan Čikoš, Lucia Olexiková, Veronika Kovaříková, Zuzana Šefčíková, Dušan Fabian

**Affiliations:** 1 Institute of Animal Physiology, Centre of Biosciences, Slovak Academy of Sciences, Šoltésovej 4-6, 040 01 Košice, Slovak Republic; 2 Research Institute for Animal Production Nitra, National Agricultural and Food Centre (NPPC), Hlohovecká 2, 951 41 Lužianky, Slovak Republic

**Keywords:** Apoptosis, Embryology

## Abstract

Apoptosis is a physiological process that occurs commonly during the development of the preimplantation embryo. The present work examines the ability of apoptotic embryonic cells to express a signal promoting their phagocytosis, and quantifies the ability of neighbouring, normal embryonic cells to perform that task. Microscopic analysis of mouse blastocysts revealed phosphatidylserine externalization to be 10 times less common than incidence of apoptotic cells (as detected by TUNEL). In spite of the low frequency of phosphatidylserine-flipping (in inner cell mass, no annexin V staining was recorded), fluorescence staining of the plasma membrane showed more than 20% of apoptotic cells to have been engulfed by neighbouring blastomeres. The mean frequency of apoptotic cells escaping phagocytosis by their extrusion into blastocyst cavities did not exceed 10%. Immunochemically visualised RAC1 (an enzyme important in actin cytoskeleton rearrangement) was seen in phagosome-like structures containing a nucleus with a condensed morphology. Gene transcript analysis showed that the embryonic cells expressed 12 receptors likely involved in phagocytic process (*Scarf1, Msr1, Cd36, Itgav, Itgb3, Cd14, Scarb1, Cd44, Stab1, Adgrb1, Cd300lf, Cd93*). In conclusion, embryonic cells possess all the necessary mechanisms for recognising, engulfing and digesting apoptotic cells, ensuring the clearance of most dying blastomeres.

## Introduction

In the early embryo, the controlled elimination of cells that are damaged or no longer needed is a normal physiological process. During the preimplantation period of development, apoptosis is usually triggered to fulfil morphogenetic functions, e.g., by regulating cell numbers or eliminating miss-positioned cells, or as part of a reparatory process, e.g., by eliminating aneuploid cells or cells damaged by internal/external factors such as cytokines, glucocorticoids, chemotherapeutic agents, oxidative stress, radiation, or the absence of growth factors^1,2^. In mammalian species, apoptotic cells become common at the morula and blastocyst stages^3,4^, and have been recorded in both the inner cell mass (ICM) and the trophectoderm (TE) cell lineages^2,5^.

It has been suggested that blastocysts eliminate dead cells via the efferocytotic action (i.e., the phagocytosis of apoptotic cells) of normal neighbouring cells^6^. Certainly, the ability to perform efferocytosis is not restricted to dedicated phagocytes such as polymorphonuclear neutrophils, monocytes and macrophages; ‘non-professional’ phagocytes such as epithelial cells, endothelial cells, fibroblasts, hepatocytes, dendritic cells and glomerular mesangial cells can also perform this function^7–9^. The phagocytic competence of early embryonic cells has been demonstrated by two in vitro studies in which 6–24 h of incubation with ≥ 1 µm particles resulted in the latters’ engulfment by trophectoderm cells in mouse^10^ and human blastocysts^11^. Random observations of phagocytosed material of apoptotic origin in pig blastomeres, as determined by transmission electron microscopy (TEM)^3^, provide further evidence that such phagocytosis occurs, as do similar observations in human^12^, cow^13^ and mouse^14^ blastocysts. Moreover, the engulfment of apoptotic bodies by neighbouring epiblast cells has recently been recorded using time-lapse imaging in mouse diploid-aneuploid mosaic chimeras cultured in vitro^15^.

The main role of efferocytosis is to prevent the release of potentially noxious or immunogenic intracellular materials and thus avoid local damage^16^. The release of degradative enzymes from the lysosomes, and of DNA which could be taken up by other cells, are potentially harmful events which have to be prevented^7^. However, while some dead cells are evidently cleared by phagocytosis within the blastocyst, others persist throughout preimplantation; indeed, isolated TUNEL + cells in the blastocoel or perivitelline space (between the trophectoderm and the zona pellucida) have been recorded in mouse and human blastocysts^6,17^. Cells that escape into lumina avoid phagocytosis^6^ and usually undergo an autolytic process known as secondary necrosis^18^. The progression of apoptosis to secondary necrosis occurs in vivo in physiological situations in which apoptotic cells are shed into areas without phagocytes (e.g., epithelial cells into the gut or airway lumen) or in pathological situations under conditions with impaired efferocytosis (e.g., when the macrophage clearance capacity is exceeded)^8,19^. Secondary necrotic cells usually decay into a mass of debris, the presence of which has been documented in blastocysts^3^. The main unresolved question is whether the frequent escape of apoptotic cells into embryonic cavities is caused by a reduced ability of apoptotic embryonic cells to express the surface markers that would promote their ingestion by neighbouring cells^6^, or by a reduced ability of these neighbouring embryonic cells to carry out efferocytosis.

The aim of the present work was to determine the physiological capacity of in vivo-developed mouse blastocysts to eliminate apoptotic cells.

## Results

### Phosphatidylserine-flipping in apoptotic embryonic cells

**Figure 1:**
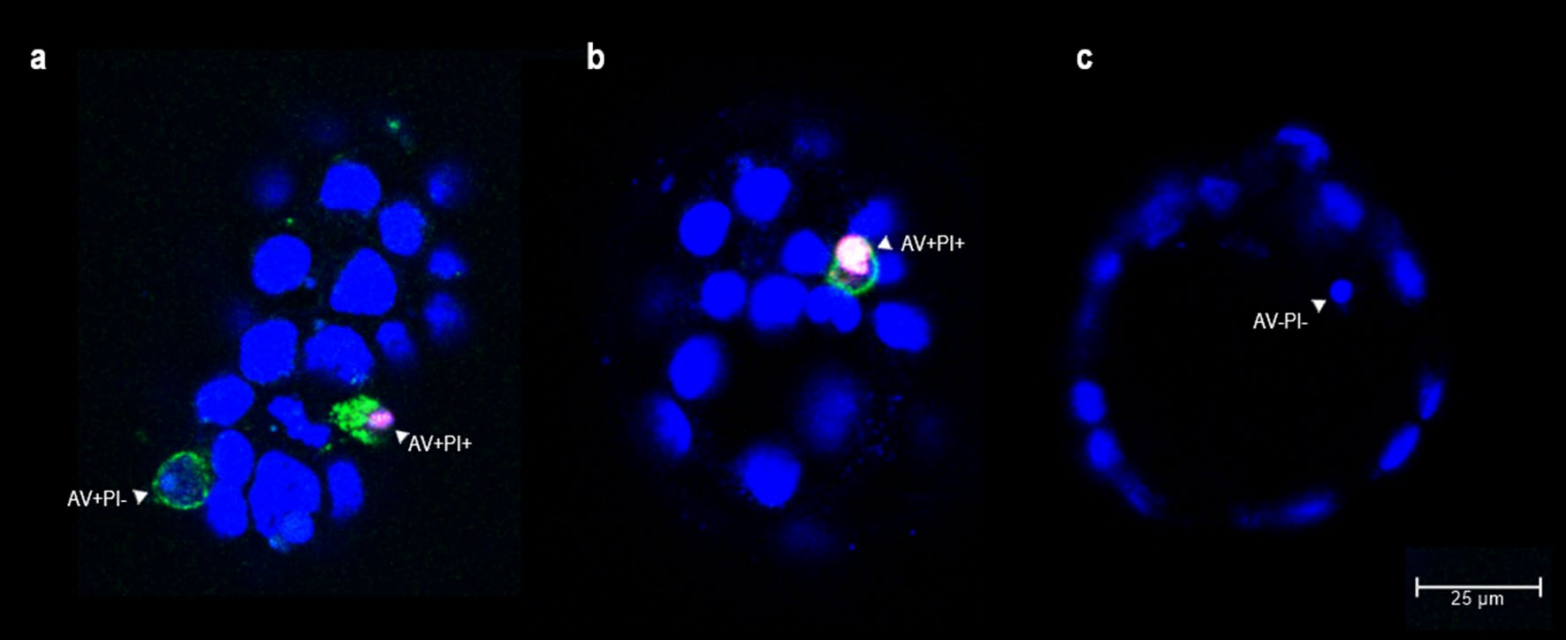
Analysis of phosphatidylserine externalization in mouse blastocysts. Images were obtained by confocal laser scanning microscopy (magnification × 400). Single optical sections of three blastocysts are shown in pictures a-c. Annexin V staining was performed to determine the presence of phosphatidylserine on the outer surface of the plasma membrane (AV +  = green); propidium iodide staining of DNA to determine cell membrane integrity (PI +  = red); and Hoechst 33342 DNA staining to visualize nuclear morphology (blue). (**a**) apoptotic AV + PI- cell showing chromatin fragmentation; secondary necrotic AV + PI + cell showing chromatin condensation; (**b**) secondary necrotic AV + PI + cell showing chromatin condensation; (**c**) apoptotic AV-PI- cell showing chromatin condensation.

To determine the ability of apoptotic embryonic cells to emit phagocytosis-promoting signals, the redistribution of phosphatidylserine (PtdSer) to the outer surface of the plasma membrane was examined. Dead cells in living mouse blastocysts were identified via the binding of fluorescein-conjugated annexin V (AV) to externalized PtdSer, and via the propidium iodide (PI) nuclear staining of cells with permeable membranes. AV and PI are commonly used in this way to monitor the progression of apoptosis^20^. Since PtdSer redistribution usually precedes morphological changes such as nuclear condensation or cell shrinkage, AV + PI- cells are considered to be at the early stages of apoptosis. AV + PI + cells are classified as late (end-stage) apoptotic cells^6^. Samples were examined using confocal laser scanning microscopy (CLSM). In 141 mouse blastocysts, 188 dead cells were identified (Table 2). However, only 2.66% showed the typical features of apoptosis, i.e., the translocation of PtdSer to the outer leaflet of the plasma membrane (AV +) and an intact nuclear envelope (PI−) (Fig. 1a). Approximately 21% of the dead cells showed PI + nuclear staining accompanied by AV + or AV- labelling; these were deemed to be secondary necrotic cells (Fig. 1a,b). None of the AV + blastomeres were located in the ICM cell lineage or blastocoele, while PI + blastomeres were observed in both the ICM and TE cell lineages and blastocyst cavities (Table 2; Supplementary Fig. S3). The remaining dead cells showed nuclear chromatin condensation only (as determined by Hoechst 33342 DNA staining [AV-PI−]) (Fig. 1c).

### Incidence of efferocytosis in mouse blastocysts

**Figure 2:**
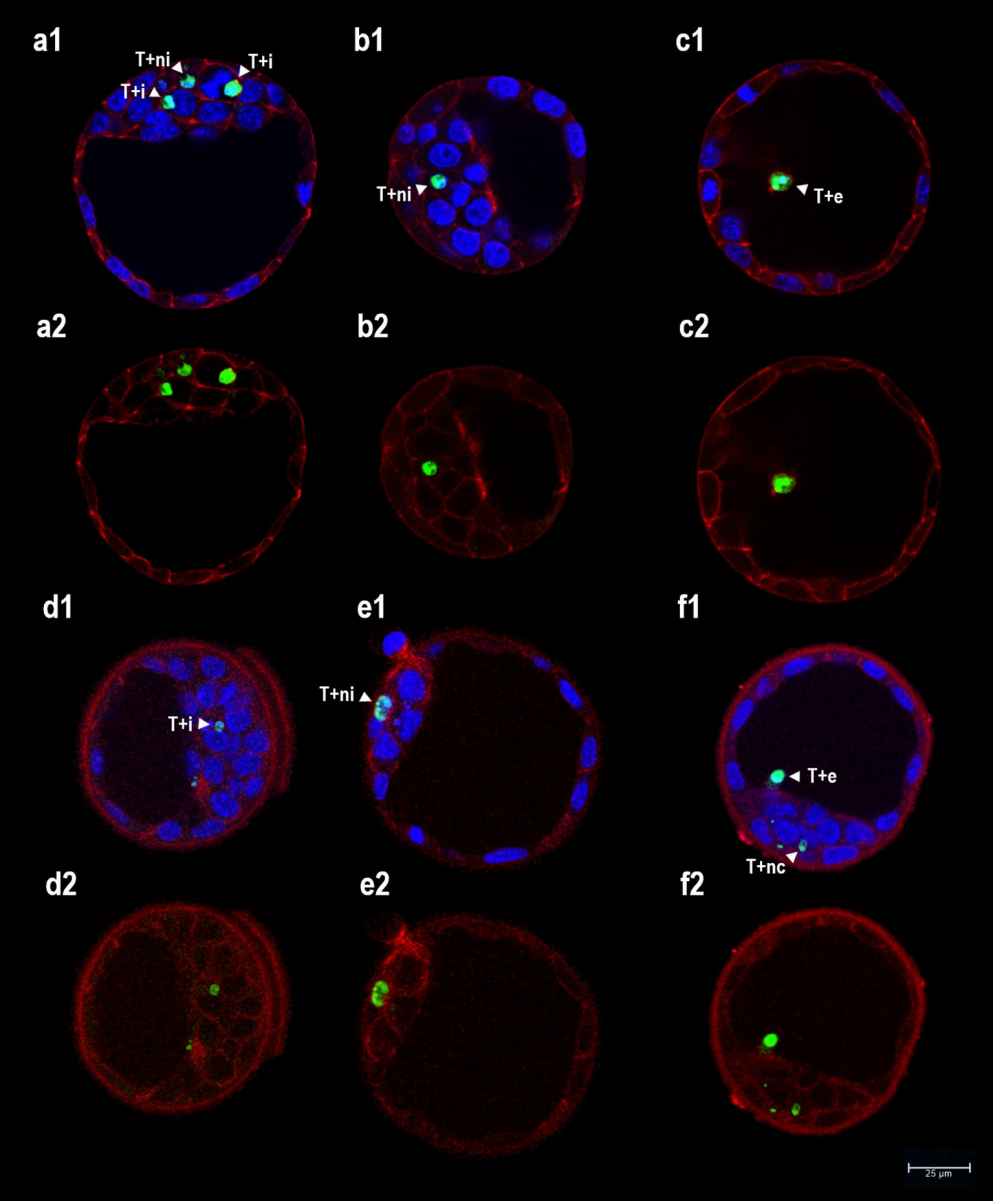
Analysis of apoptotic cell internalization in mouse blastocysts. Images were obtained by confocal laser scanning microscopy (magnification × 400). Single optical sections of six blastocysts are shown in pictures (**a–f**). (**a–c**) fluorescence staining of F-actin in plasma membrane (red) combined with TUNEL labelling (T +  = green) and Hoechst 33342 DNA staining (blue) (**a1**–**c1**: merge); (**d**–**f**) immunochemical staining of E-cadherin in plasma membrane (red) combined with TUNEL labelling (T +  = green) and Hoechst 33342 DNA staining (blue) (**d1**–**f1**: merge). Abbreviations: i, internalized apoptotic nuclei (T + nuclei in embryonic cells with a normal nucleus and intact plasma membrane); ni, non-internalized apoptotic nuclei (T + nuclei surrounded by the intact plasma membranes of at least three neighbouring cells); e, extruded apoptotic nuclei (in blastocoele); nc, non-classified apoptotic nuclei (dubious cases).

**Figure 3:**
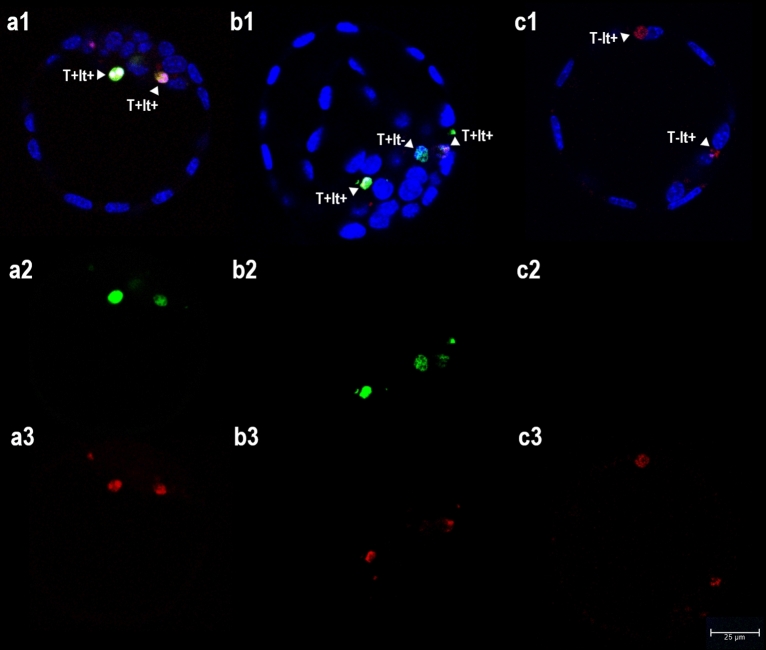
Analysis of acid organelles in mouse blastocysts. Images were obtained by confocal laser scanning microscopy (magnification × 400). Single optical sections of three blastocysts are shown in pictures (**a–c**). To determine the presence of acid organelles (lysosomes/phagosomes) in embryonic cells, LysoTracker labelling was used (lt +  = red, **a3**–**c3**), combined with TUNEL labelling (T +  = green, **a2**–**c2**) and Hoechst 33342 DNA staining (blue) (**a1**–**c1**: merge). (**a**) Two apoptotic nuclei with condensed chromatin showing co-localization with acid organelles (T + lt+); (**b**) two apoptotic nuclei with condensed/fragmented chromatin showing co-localization with acid organelles (T + lt+) and one TUNEL + nucleus with normal morphology without acid organelles (T + lt−); (**c**) two normal nuclei showing co-localization with acid organelles (T-lt+).

The effectiveness of apoptotic cell elimination was examined by visualizing the internalization/extrusion of such cells. Apoptotic cells were identified in fixed mouse blastocysts via the combination of TUNEL (to detect the presence of DNA fragmentation) and fluorescence DNA staining. The latter reveals the morphological features of the nucleus, including chromatin condensation and karyorrhexis, i.e., features typical of the execution phase of apoptosis^20^. In 168 mouse blastocysts, 772 dead cells were identified (Table 2). TUNEL revealed 95.92% of cells with condensed nuclear chromatin (usually decayed into smaller fragments) to show specific DNA fragmentation in the nucleoplasm as well. Such cells were classified as apoptotic (Figs. 2, 3). Rarely, TUNEL + labelling was observed in cells with normal nuclear morphology (Fig. 3b1, Table 2). A larger number of cells with chromatin condensation and/or TUNEL + labelling was recorded in the ICM and blastocoele than in the TE and perivitelline space (Mann–Whitney U test, *P* < 0.0001; Fig. 2). In general, blastocysts subjected to TUNEL showed significantly higher numbers of dead cells than did blastocysts subjected to AV/PI staining (Mann–Whitney U test, *P* < 0.0001; Table 2).

The internalization of apoptotic cells was assessed by fluorescence staining of F-actin and the immunochemical staining of E-cadherin. As shown in Table 3, similar results were obtained in different groups of blastocysts subjected to these assays (Chi-squared test, *P* = 0.056): 20–26% of TUNEL + nuclei were internalized in the cytoplasm of neighbouring embryonic cells (Fig. 2a,d); around 9.5% of TUNEL + nuclei had become extruded into the blastocoele or perivitelline space (Fig. 2c,f); and 27–34% of TUNEL + nuclei were not internalized (i.e., they were surrounded by the intact plasma membranes of at least three neighbouring cells) (Fig. 2b,e). The remaining TUNEL + nuclei were not classified (Fig. 2f) due to the degradation of F-actin and E-cadherin in the plasma membrane. Internalization of apoptotic cells was observed in both the ICM and TE cell lineages.

To examine phagosome formation, two assays were performed: acid organelle tracking using the LysoTracker kit and immunochemical staining of the active form of RAC1 (Rac Family Small GTPase 1), an enzyme important in actin cytoskeleton rearrangement during the engulfment of apoptotic cells^21^.

In the case of LysoTracker staining, the proportion of TUNEL + nuclei co-localized with lysosomes reached 95.80% (Table 3, Fig. 3). Unfortunately, the relatively low volume of cytoplasm in embryonic cells allowed for no distinction between apoptotic nuclei internalized in the phagosomes of neighbouring cells and non-internalized apoptotic cells with accumulated lysosomes in the cytoplasm (at least when viewed under CLSM). LysoTracker staining was also observed in apoptotic cells that had become extruded into the blastocoele and perivitelline space.

**Figure 4:**
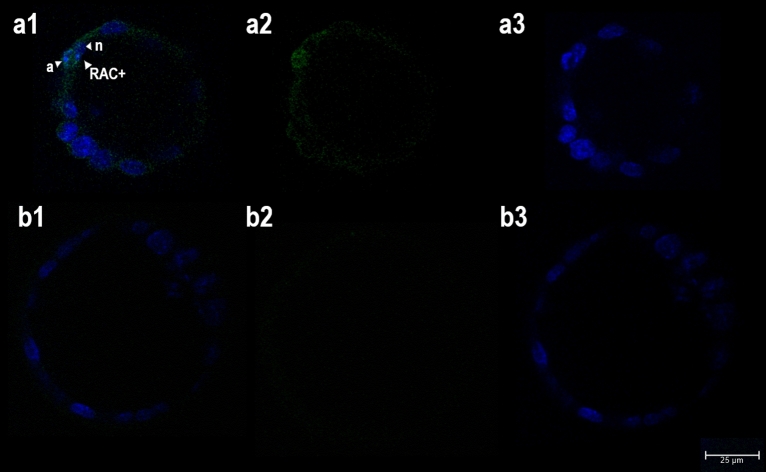
Analysis of phagosome formation in mouse blastocysts. Images were obtained by confocal laser scanning microscopy (magnification × 400). Single optical sections of two blastocysts are shown in the pictures (**a,b**). (**a**) Immunochemical staining of RAC1 (**a2**, green) combined with Hoechst 33342 DNA staining (**a3**, blue) (**a1**: merge). (**b**) Negative control staining (**b2**, omission of primary antibody) combined with Hoechst 33342 DNA staining (**b3**, blue) (**b1**: merge). (**a1**) presence of RAC1 (RAC +) in a membrane-like formation surrounding one nucleus with a condensed morphology (a) next to nucleus with normal morphology (n).

A weak signal for RAC1 was seen in the cytoplasm of the majority of embryonic cells (Fig. 4a). However, among the 56 blastocysts examined, only five showed a particularly strong signal. In three, RAC1 was present in phagosome-like structures surrounding nuclei with a condensed morphology (Fig. 4a1), in a one it was seen around a nucleus with condensed morphology and around a nucleus with normal morphology, and in one it was detected around a nucleus with normal morphology (with no apoptotic cells in the neighbourhood) (data not shown).

**Figure 5:**
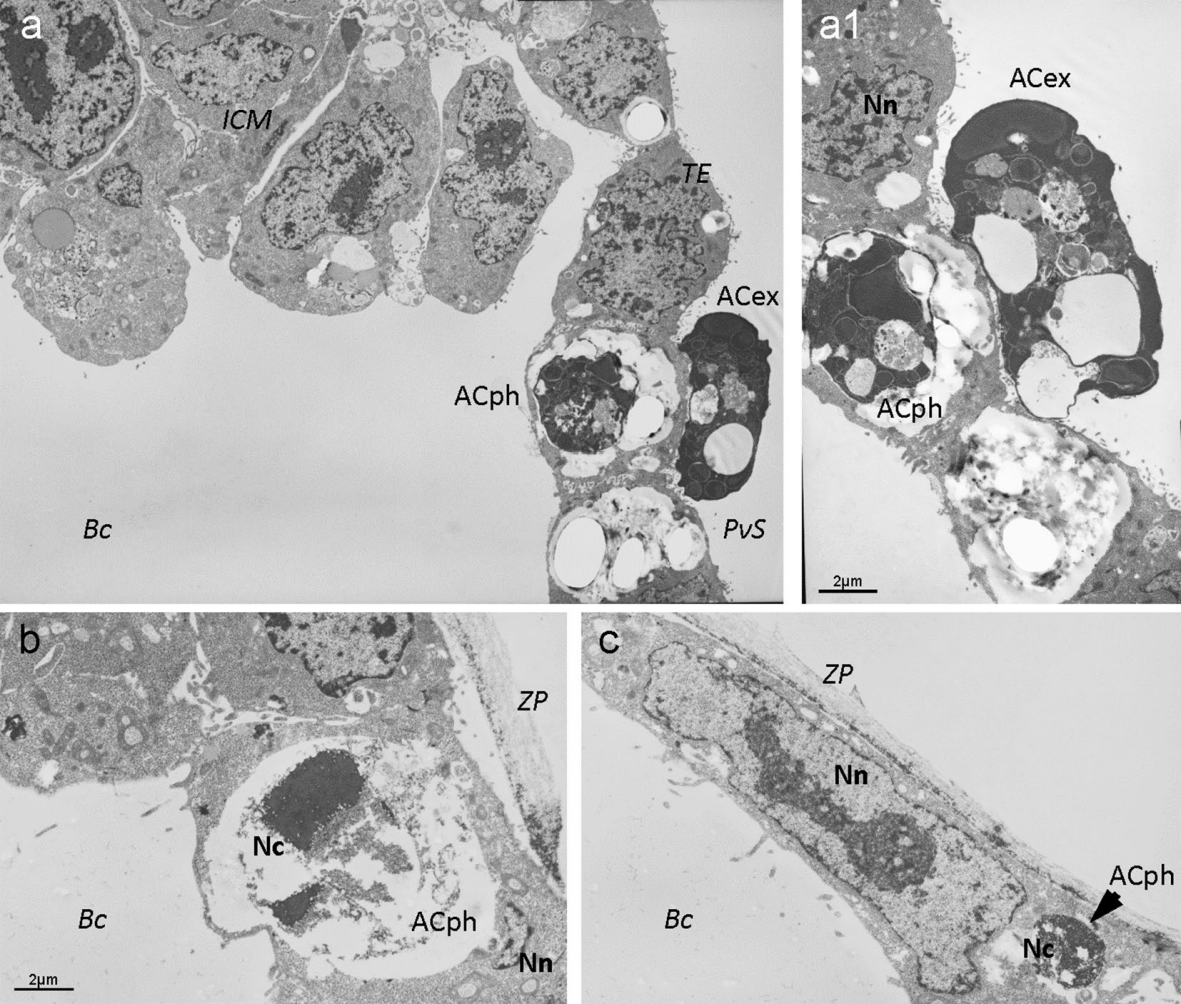
Ultrastructural analysis of efferocytosis in mouse blastocysts. Images were obtained by transmission electron microscopy. Sections of three blastocysts are shown in pictures (**a–c**). (**a**) Electron micrograph showing the localisation of the inner cell mass (ICM), trophectoderm (TE), perivitelline space (PvS) and blastocoel (Bc); (**a1**) a detail of dead cell with dense cytoplasmic matrix phagocytosed by a normal TE cell (ACph) and of a dead cell showing typical features of apoptosis (shrinkage, higher density of cytoplasmic matrix, crowding of organelles, chromatin condensation and decay into fragments, and increased number of vacuoles) extruded to the PvS (ACex); (**b**) electron micrograph of a dead cell with condensed chromatin (Nc) and highly translucent (lysed) cytoplasmic matrix (ACph) phagocytosed by a normal TE cell with normal nuclear chromatin (Nn) enclosed by zona pellucida (ZP); (**c**) electron micrograph of a dead cell with condensed chromatin (Nc) phagocytosed by a normal TE cell with normal nuclear chromatin (Nn) enclosed by zona pellucida (ZP).

The ultrastructural assessment of mouse blastocysts using TEM also revealed the presence of phagosomes in embryonic cells (Fig. 5). These phagosomes contained remnants of dead cells at different stages of decomposition. Some of phagocytosed cells showed chromatin condensation or chromatin decay into fragments, the crowding of organelles, an increased number of vacuoles, and a higher density of cytoplasmic matrix (Fig. 5a1); other phagocytosed cells showed chromatin condensation and a highly translucent cytoplasmic matrix (Fig. 5b,c).

### Expression of selected receptors and immunoregulatory proteins involved in efferocytosis

The expression of phagocyte receptors and “bridge” proteins in mouse blastocysts was evaluated using RT-PCR (Table 1) in order to assess the ability of embryonic cells to initiate efferocytosis. The main criterion for the selection of genes was the previously documented involvement of their transcripts in the clearance of apoptotic cells in other types of non-professional phagocytes (Supplementary Table S1).

**Table 1:** Genes, oligonucleotide primers and PCR product lengths (*Mus musculus*).

Gene symbol	Gene description	RefSeq no.	Qiagen product no.	PCR product length (bp)
*Cd36*	CD36 molecule (Scavenger receptor B3)	NM_007643	PPM03796D	187
*Stab1*	Stabilin 1	NM_138672	PPM33734A	137
*Scarf1*	Scavenger receptor class F, member 1	NM_001004157	PPM25600A	89
*Itgav*	Integrin alpha V	NM_008402	PPM03662D	66
*Itgb3*	Integrin beta 3	NM_016780	PPM03687E	67
*Cd14*	CD14 antigen	NM_009841	PPM06249G	170
*Adgrb1*	Adhesion G protein-coupled receptor B1	NM_174991	PPM05646E	84
*Cd300lf*	CD300 molecule like family member F	NM_145634	PPM37067B	100
*Msr1*	Macrophage scavenger receptor 1	NM_031195	PPM03795A	117
*Scarb1*	Scavenger receptor class B, member 1	NM_016741	PPM05368C	157
*Cd44*	CD44 antigen	NM_009851	PPM03628F	88
*Cd93*	CD93 antigen (C1q receptor)	NM_010740	PPM24562A	191
*Sftpa1*	Surfactant associated protein A1	NM_023134	PPM05286A	160
*Mbl1*	Mannose-binding lectin (protein A) 1	NM_010775	PPM37319A	123
*Mbl2*	Mannose-binding lectin (protein C) 2	NM_010776	PPM27711A	172

RefSeq No., GenBank reference sequence accession number; Bp, base pairs; Qiagen product No., Product numbers of Qiagen Primer Assays.

**Figure 6:**
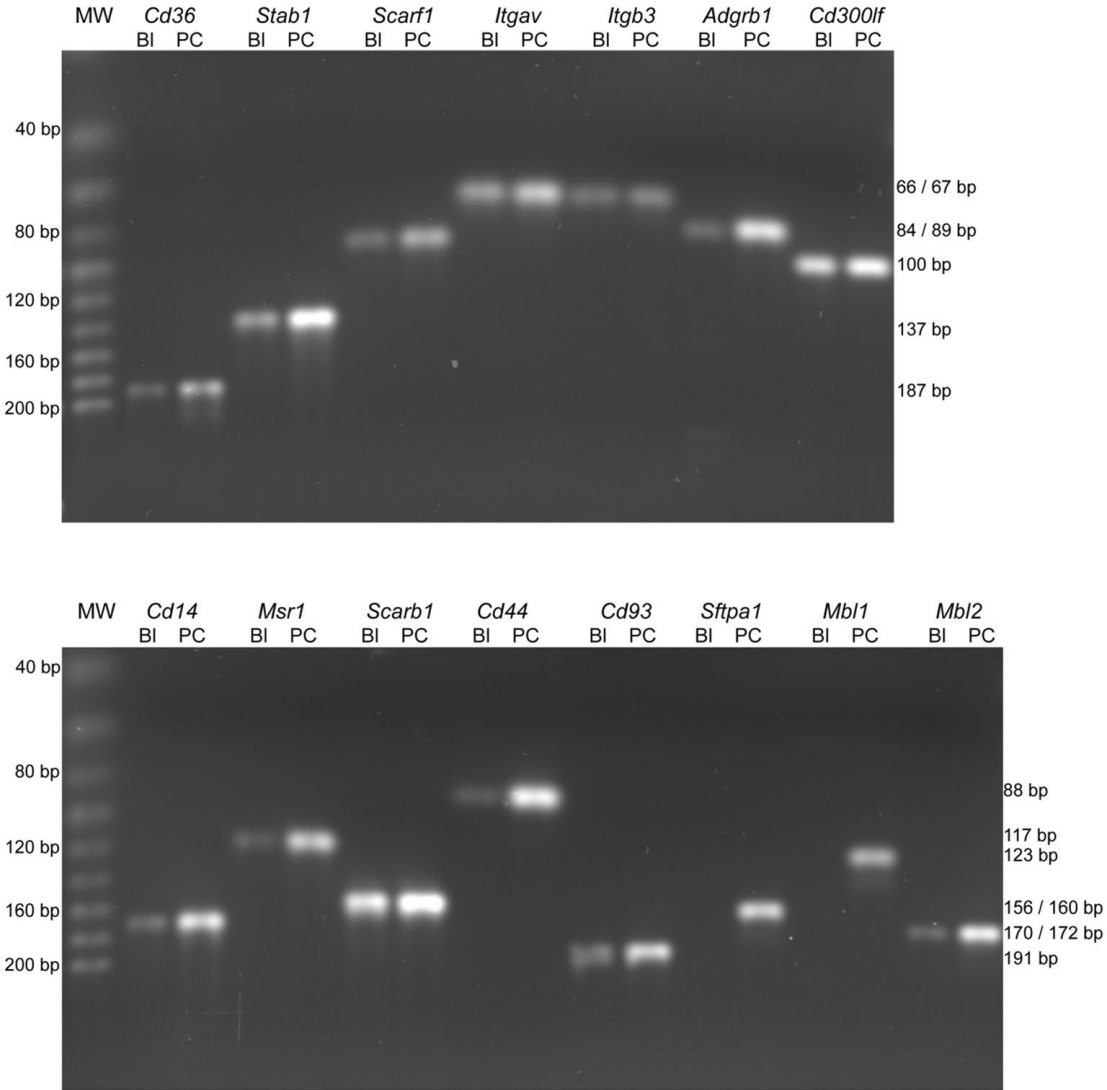
RT-PCR analysis of selected receptors and immunoregulatory proteins in mouse blastocysts. Representative agarose gels with separated PCR products are shown. Lanes: MW, molecular weight markers; *Cd36*, CD36 antigen; *Stab1*, stabilin 1; *Scarf1*, scavenger receptor class F, member 1; *Itgav*, integrin alpha V; *Itgb3*, Integrin beta 3; *Adgrb1*, adhesion G protein-coupled receptor B1; *Cd300lf*, CD300 antigen like family member F; *Cd14*, CD14 antigen; *Msr1,* macrophage scavenger receptor 1; *Scarb1,* scavenger receptor class B, member 1; *Cd44,* CD44 antigen; *Cd93,* CD93 antigen; *Sftpa1*, surfactant associated protein A1; *Mbl1,* mannose-binding lectin (protein A) 1; *Mbl2,* mannose-binding lectin (protein C) 2; Bl, blastocyst; PC, positive control tissue. The MWs and the predicted sizes of the PCR products in base pairs (bp) are indicated to the left and right of the panels respectively.

The reaction conditions were optimized for each primer pair in positive control tissues before using experimental samples. PCR products corresponding to transcripts of almost all examined genes were detected in the blastocysts (Fig. 6). For two genes, *Mbl1* and *Sftpa1*, the corresponding PCR products were detected only in positive control tissues but not in blastocysts (Fig. 6). Blastocysts samples produced a PCR fragment corresponding to β-actin mRNA, confirming the integrity of the RNA and the efficiency of the RT-PCR process (data not shown). No specific PCR products were detected in the reactions in which cDNA was omitted (blank reactions, see Supplementary Fig. S1).

## Discussion

### Phosphatidylserine-flipping in apoptotic embryonic cells

During apoptosis PtdSer translocates to the outer leaflet of the membrane bi-layer. Here it is recognized by phagocytes carrying appropriate membrane-bound receptors, some of which require the assistance of soluble “bridge” molecules to finally attach to the PtdSer^8,9^. The existence of other marker molecules (e.g., altered sugars recognized by lectins), plus PtdSer-independent signalling pathways for the induction of engulfment of apoptotic cells, have been proposed, but they remain to be fully characterized^22,23^.

The present results confirm previous findings that mouse embryonic cells redistribute PtdSer to the outer surface of the plasma membrane during apoptosis. However, compared to the overall frequency of apoptosis (as determined by TUNEL), this process was little seen (4.34 ± 3.46 TUNEL + cells with condensed chromatin per blastocyst vs. 0.04 ± 0.19 AV + PI− cells per blastocyst; Mann–Whitney U test, *P* < 0.0001). Positive AV staining was also recorded in 2/3 of PI-labelled cells (0.28 ± 0.55 per blastocyst), which probably represented apoptotic cells undergoing secondary necrosis. However, the overall frequency of PtdSer-flipping in the membrane of dying cells was still > 10 times lower than that of DNA fragmentation in the nucleoplasm (Table 2).

**Table 2:** Frequency of embryonic cells showing features of apoptosis or secondary necrosis.

Staining	AV/PI staining		TUNEL assay	
	Total n (%)	Aver. n per blastocyst	Total n (%)	Aver. n per blastocyst
Evaluated mouse blastocysts	141		168	
Blastocysts containing dead cells	92 (65.25%) ^a^		148 (88.10%) ^b^	
Evaluated dead cells	188	1.33 ± 1.51 ^a^	772	4.60 ± 3.46 ^b^
*Dead cells localized in ICM (or Bc)*	*129*	*0.91* ± *1.17 *^*a*^	*538*	*3.20* ± *2.81 *^*b*^
*Dead cells localized in TE (or PvS)*	*59*	*0.42* ± *0.63 *^*a*^	*234*	*1.39* ± *1.59 *^*b*^
*ICM:TE distribution*	*(68.62:31.38%)*^a^		*(69.69:30.31%)*^a^	
AV-/PI- cells with condensed nuclei	144 (76.60%)	1.02 ± 1.52	n/a	n/a
*ICM:TE distribution*	*120:24 (83.33:16.67%)*		*n/a*	
AV + /PI- cells	5 (2.66%)	0.04 ± 0.19	n/a	n/a
*ICM:TE distribution*	*0:5 (0.00:100.00%)*		*n/a*	
AV + /PI + cells	26 (13.83%)	0.18 ± 0.42	n/a	n/a
*ICM:TE distribution*	*0:26 (0.00:100.00%)*		*n/a*	
AV-/PI + cells	13 (6.91%)	0.09 ± 0.38	n/a	n/a
*ICM:TE distribution*	*9:4 (69.23: 30.77%)*		*n/a*	
T + nuclei with apoptotic morphology*	n/a	n/a	729 (94.43%)	4.34 ± 3.46
T + nuclei with normal morphology	n/a	n/a	12 (1.55%)	0.07 ± 0.32
T- nuclei with apoptotic morphology*	n/a	n/a	31 (4.02%)	0.18 ± 0.61

Results are expressed as arithmetical means ± standard deviation or percentages. AV, annexin V; PI, propidium iodide; T, TUNEL labelling; ICM, inner cell mass; Bc, blastocoel; TE, trophectoderm; PvS, perivitelline space; *apoptotic morphology: condensed nuclear chromatin usually decayed into smaller fragments. Different superscript letters indicate significant differences. Statistical analysis: Proportion of blastocysts containing dead cells: Chi-squared test with one degree of freedom (*P* < 0.0001); mean number of evaluated dead cells per blastocysts: Mann–Whitney U test (*P* < 0.0001 for all cases); ICM:TE distribution: Chi-squared test with one degree of freedom (*P* = 0.791).

To rule out the possibility that this disproportion was caused by some methodological error, two commercial staining kits from different sources were used to evaluate PtdSer externalization; in both cases the results were similar.

To our knowledge, the frequency of PtdSer-flipping in mouse in vivo-developed blastocysts has not previously been examined. So far, only data on the frequency of AV + cells in in vitro derived blastocysts have been published: Mouse Day 5 blastocysts developed from two-cell embryos showed no AV + signal^24^, even after short-time vitrification^25^. An AV + signal was recorded in approximately 15% of Day 5 blastocysts developed from naturally fertilized zygotes^26^. Freshly isolated blastocysts subjected to 24 h culture in vitro showed around 1% AV + PI− cells, i.e., approx. 1.6 AV + cells per blastocyst^27–29^. In the latter works the frequency of AV + cells was higher than that observed in the present study; however, the difference is probably due to a general increase in the frequency of apoptosis in in vitro cultured embryos and in older blastocysts (Day 6 vs. Day 4 in the present work). Relatively high frequencies of PtdSer-flipping have also been reported in bovine Day 6 blastocysts^30^ and porcine Day 7 blastocysts^31^ derived from in vivo-fertilized oocytes and in vitro-cultured embryos.

Despite these numerical differences, apparently due to species specificity, the method of blastocyst production and the stage of blastocyst development, the majority of studies report a frequency of AV + blastomeres significantly lower than the frequency of TUNEL + blastomeres. There are several possible explanations for this:

1. The absence of an AV + signal in ICM cells (Table 2) recorded in the present work suggests that the ability of conjugated AV (35–36 kDa) to pass through tight junction pores of the TE layer in living blastocysts is extremely low. (It has been shown that tight junctions act as seals that are impermeable to certain molecules with a molecular weight of > 4 kDa^32^.)
2. It is possible that no externalized PtdSer was detected in some embryonic apoptotic cells because they had probably already undergone phagocytosis by the time of assay^33^. (In vivo studies of differentiated tissues have shown that the uptake of dying cells by their phagocytic neighbours might occur early in apoptosis, even before DNA fragmentation^7^.)
3. Some portion of TUNEL + cells in the present study should probably not be classified as apoptotic because of the relatively low specificity of the TUNEL assay for detecting apoptosis. (It is known that the production of DNA fragments with 3′-OH ends can be related to other forms of DNA damage, and the TUNEL assay certainly detects DNA breaks associated with necrotic cell death and active DNA repair^34,35^.)

Finally, previously postulated hypothesis on poorer ability of embryonic apoptotic cells to externalize PtdSer cannot be excluded as well^6^. However, since PtdSer-flipping is based on a relatively simple mechanism (caspases 3 and 7 irreversibly deactivate the flippases ATP11A and ATP11C, and in the absence of flippases, the PtdSer remains exposed and does not return to the inner leaflet^36^), we assume its disruption the least likely.

### Incidence of efferocytosis in mouse blastocysts

Several efferocytosis assays have been standardized for in vitro or in vivo studies of professional phagocytes. In order to monitor the process, target cells are usually labelled with an apoptosis marker or cell tracker, and phagocytes are labelled with a pH sensitive dye or membrane stains. A similar approach has been used for the morphological assessment of efferocytosis in non-professional phagocytes, with specific modifications^37^.

In the present work, apoptotic cells in mouse blastocysts were visualized by TUNEL (combined with morphological chromatin staining), and matured (acidified) phagosomes in embryonic phagocytes by LysoTracker labelling. CLSM revealed that almost 96% of TUNEL + nuclei were co-localized with acid organelles (Table 3). However, it was impossible to distinguish between apoptotic cells internalized in phagosomes by neighbouring cells and non-internalized apoptotic cells with accumulated lysosomes in the cytoplasm (Fig. 3).

**Table 3:** Analysis of efferocytosis in mouse blastocysts using different types of fluorescence staining.

Staining	F-actin (phalloidin-T)		E-cadherin (IHC)		Lysotracker	
Target organelle	Plasma membrane		Plasma membrane		Acid organelles (lysosomes)	
	Total n (%)	Av. n per blastocyst	Total n (%)	Av. n per blastocyst	Total n (%)	Av. n per blastocyst
Evaluated mouse blastocysts	56		56		56	
Blastocysts containing T + nuclei	56 (100.00%)		44 (78.57%)		48 (85.71%)	
Evaluated T + nuclei	247	4.41 ± 2.54 ^ab^	192	3.43 ± 2.88 ^a^	333	5.95 ± 4.28 ^b^
Internalized T + nuclei	64 (25.91%)	1.14 ± 1.26 ^a^	39 (20.31%)	0.70 ± 0.99 ^b^	n/a	n/a
Non-internalized T + nuclei	83 (33.60%)	1.48 ± 1.48 ^a^	51 (26.56%)	0.91 ± 1.08 ^b^	n/a	n/a
Extruded T + nuclei	23 (9.31%)	0.41 ± 0.65 ^a^	19 (9.90%)	0.34 ± 0.82 ^a^	n/a	n/a
Non-classified T + nuclei	77 (31.18%)	1.38 ± 1.42 ^a^	83 (43.23%)	1.48 ± 1.57 ^a^	n/a	n/a
T + nuclei co-localized with acid organelles	n/a	n/a	n/a	n/a	319 (95.80%)	5.70 ± 4.21
T + nuclei without acid organelles	n/a	n/a	n/a	n/a	14 (4.20%)	0.25 ± 0.64

Results are expressed as arithmetical means ± standard deviation or percentages; T + , TUNEL positive labelling. Different superscript letters indicate significant differences. Statistical analysis: mean number of evaluated TUNEL + nuclei: Kruskal–Wallis test (*P* = 0.003), followed by Dunn’s test; mean number of internalized TUNEL + nuclei: Mann–Whitney U test (*P* = 0.048); mean number of non-internalized TUNEL + nuclei: Mann–Whitney U test (*P* = 0.039); mean number of extruded TUNEL + nuclei: Mann–Whitney U test (*P* = 0.312); mean number of non-classified TUNEL + nuclei: Mann–Whitney U test (*P* = 0.874); Proportion of internalized, non-internalized, extruded and non-classified TUNEL + nuclei: Chi-squared test with three degrees of freedom (*P* = 0.056).

To visualize the internalization of apoptotic cells, two methods of membrane staining were used: direct fluorescence staining of F-actin and the immunochemical staining of E-cadherin. Although the outcomes of proportional analysis were similar (around 23% of indisputably internalized and around 39% of clearly non-internalized apoptotic cells), the fluorescence staining option was accompanied with easier classification: after direct membrane visualization with phalloidin-TRITC, the signal was very regular and the numbers of identified internalized and non-internalized TUNEL + nuclei were significantly higher (Table 3). Unfortunately, in both assays the proportion of non-classified apoptotic cells was quite high (over 30%). This was because most of the apoptotic cells showed no membrane staining (Fig. 2). Although the integrity of cell membranes remains intact until the very late stages of apoptosis^38^, selected membrane and cell membrane-associated proteins are apparently cleaved away relatively early.

To overcome the problem with the classification of apoptotic cells with unstained membranes, the visualization of membrane lipids was attempted using carbocyanine staining. However, despite much effort, the protocol for the quantitative analysis of apoptotic cell internalization could not be standardized (see Supplementary Fig. S2).

To obtain more information on phagosome formation in mouse blastocysts, the immunochemical evaluation of RAC1 in its active form was undertaken. RAC1 activation is necessary for F-actin assemblage, and its de-activation is obligatory for closing the phagocytic cup^39^. Studies on professional phagocytes have shown RAC1 to be involved in a highly-conserved signalling pathway downstream from the most common PtdSer receptors^40^.

The expression of RAC1 has been confirmed in numerous tissues, including mouse and porcine oocytes and porcine parthenogenic embryos^41,42^. The RAC1 localization pattern in porcine oocytes and embryos was found to be similar to that of the actin filaments: it accumulated exclusively at the cortex of oocytes and blastomeres of cleavage stage-embryos. An analogous RAC1 distribution was recorded in the mouse blastocysts in the present study (Fig. 4). The presence of the enzyme in membrane-like formations surrounding several nuclei with condensed morphology indicates that RAC1 plays an important role in the engulfment of apoptotic cells during preimplantation development. Compared to other efferocytosis assays, the frequency of pertinent observations was very low. However, this might be explained by the rapidity of the engulfment process, i.e., the very narrow window for detecting it.

Taken together, the present results show that, in spite of the relatively low frequency of PtdSer externalization detected, embryonic cells clear up apoptotic cells by phagocytosis. Since the numeric outcomes of the efferocytosis assays differed, it is not possible to define an exact efferocytotic index. However, the mean number of apoptotic cells that escaped phagocytosis can be estimated. Of all the evaluated apoptotic cells (mean 3.43 or 4.41 cells per blastocyst depending on staining group [Table 3]), only 10% were extruded into blastocyst cavities (0.34 or 0.41 per blastocyst [Table 3]) and around 6% underwent secondary necrosis (0.28 per blastocyst [AV ± PI + , Table 2]). Since there was an overlap (a portion of secondary necrotic cells was localized in embryonic cavities, especially the perivitelline space) it might be concluded that the proportion of dying cells with non-phagocytosed status exceeded no more than 15% (i.e., approx. one cell in every two blastocysts). Thus, the overall frequency of efferocytosis in mouse blastocysts might be deemed relatively high.

### Expression of selected receptors and immunoregulatory proteins involved in efferocytosis

No single dominant phagocyte receptor has ever been identified as responsible for apoptotic cell clearance. Previous studies have revealed many different and often unrelated phagocyte receptors for apoptotic cells. Moreover, in experimental systems, the complete inhibition of efferocytosis has never been achieved, even when inhibitory antibodies or ligands have been used in combination^8,43^. Thus, the recognition of apoptotic cells appears to be a cooperative event involving several receptors that come into play simultaneously or sequentially^7^.

Only a few cell surface receptors, such as TIM-1, TIM-3, TIM-4, stabilin 2, ADGRB1 and CD300LF, directly recognize PtdSer. Other receptors can specifically bind to PtdSer through soluble bridging proteins such as lactadherin, calreticulin, or thrombospondin (e.g. αVβ3 integrin [vitronectin receptor], LRP1 receptor [low density lipoprotein receptor-related protein 1])^9,21,44,45^.

Apoptotic cells can also be recognized by scavenger receptors. Some scavenger receptors, such as CD36, can bind various anionic phospholipids including PtdSer. However, it is likely that the oxidation, acetylation or glycosylation of lipoproteins in apoptotic or secondary necrotic cells generates a much wider range of ligands for scavenger receptors. In addition to CD36 (scavenger receptor class B, member 3), involvement in efferocytosis has been demonstrated for scavenger receptors A, B1, and F1^7,40,46^. Naturally (and as reviewed in several studies), the list of efferocytosis receptors is much longer^8,19,21,33,47^.

In addition to the surface receptors of phagocytes, specific immunoregulatory proteins, such as collectins, are important in the clearance of apoptotic and necrotic cells. Collectins (e.g. mannose-binding lectins [MBL]) primarily recognize pathogen-associated molecular patterns by binding to their sugar moieties. Structural and functional homology with the collectins is also shared by C1q, a member of the first component of the classical complement pathway^48,49^.

The function of the listed molecules has been predominantly studied in professional phagocytes. Certainly, the expression of several efferocytosis receptors has also been documented in non-professional phagocytes, such as endothelial cells (sinusoidal and kidney), hepatocytes, retinal pigment cells and fibroblasts^33^, but no data are available on their expression in embryonic cells. To our knowledge, only transcripts of PtdSer receptor have been evaluated in embryos. One knock-out study has shown that this receptor is essential for the development and differentiation of multiple organs during embryogenesis, but not for apoptotic cell removal^16^.

The present study documents the expression of 12 receptors and one bridging protein which could be involved in the phagocytosis of apoptotic blastomeres (Fig. 6). As expected, the mouse embryonic cells expressed CD36 receptor, previously shown to be involved in the clearance of apoptotic cells in both professional and non-professional human phagocytes^50,51^. A gene homologous to mammalian CD36 has also been found in the genome of *Caenorhabditis elegans* and *Drosophila melanogaster*, which suggests its strong conservation in the molecular machinery of efferocytosis^7^.

The present results also reveal the embryonic transcription of another four receptor genes previously found to be expressed in mouse or human endothelial cells (STAB1, SCARF1 and CD14) and human fibroblasts (vitronectin receptor). Certainly, stabilin 1 (STAB1) can directly recognize PtdSer^52^, and scavenger receptor class F member 1 (SCARF1) and vitronectin receptor (integrin αVβ3) usually bind to PtdSer through soluble bridging proteins^44,53^*.* Surprisingly, the present mouse embryonic cells also expressed (at least at the mRNA level) six efferocytosis receptors previously described only in human macrophages or mouse myelocytes: adhesion G protein-coupled receptor B1 (ADGRB1) and CD300 molecule-like family member F (CD300LF), which bind directly to PtdSer^45,54^; macrophage scavenger receptor 1 (SCARA1) and scavenger receptor B1 (SCARB1), which bind to oxidized lipids^46^; the CD44 receptor, which binds to various ligands including hyaluronic acid, osteopontin, collagens and matrix metalloproteinases^55^; and the CD93 receptor, which binds to moesin and C1q^48^. However, only one of three evaluated regulatory proteins for efferocytosis was detectable, i.e., mannose-binding lectin 2 (MBL2), which has previously been shown to assist in the engulfment of apoptotic cells by human macrophages^48^. That said, no transcripts for mannose-binding lectin 1 (MBL1) or surfactant associated protein A1 (SFTPA1) were found in the present blastocysts.

Taken together, mouse embryonic cells would appear to be equipped with a wide range of receptors necessary for the recognition of apoptotic cells. However, some of them are of pleiotropic functions: scavenger receptors might be also engaged in engulfment of lipoproteins or lipid metabolism^56^, others might play important role during implantation of blastocyst (e.g. integrin αVβ3^57^, CD44^58^). Thus, their specific role and importance in early embryonic efferocytosis remains to be determined.

## Conclusions

The present study provides the first insight into the machinery of embryonic efferocytosis and quantitative analysis of its efficiency.

Results show that embryonic cells in mouse blastocysts possess all the mechanisms necessary for the recognition, engulfment and digestion of damaged blastomeres. The process of embryonic efferocytosis seems to follow the standard pathway: It begins with the recognition of the apoptotic cell via the binding of various phagocytic receptors to externalized PtdSer (or other ligands such as modified lipoproteins). Signalling leads to the recruitment of Rho family GTPase RAC1, the actin-dependent formation of the phagocytic cup and targeted internalization, i.e., taking the apoptotic cell into a vacuole (the phagosome) which then undergoes maturation (progressive acidification) and gradual degradation.

It would thus appear that intact early embryonic cells can act as non-professional phagocytes and undertake the clearance of the majority of dying cells in blastocysts. Since the numbers of blastomeres escaping efferocytosis are relatively low (up to one cell in every two blastocysts), it might be hypothesized that the main reason for cell extrusion in mouse blastocysts is neither the reduced ability of apoptotic cells to express markers promoting their ingestion nor a reduced ability of neighbouring embryonic cells to engulf them, but simply the loss of contact between normal and apoptotic cells caused by the condensation of the latters’ content and their overall shrinkage.

Finally, the preimplantation embryo appears to be a unique experimental model for studying the physiology of efferocytosis. The blastocyst represents a comprehensive in vivo system consisting of pluripotent cells with a specific phylogenetic position between the invertebrates and adult mammals with developed immune systems. The evaluation of efferocytosis characteristics in embryos could provide further information on key evolutionary-conserved patterns in this process.

## Methods

Unless otherwise indicated, all chemicals were purchased from Sigma-Aldrich (Saint-Louis, Missouri, USA).

### Animals and embryo recovery

Female mice (30–35 days old) underwent hormonal synchronization using pregnant mare's serum gonadotropin (eCG, 5 IU intraperitoneally) (Folligon, Intervet International, Boxmeer, Holland), followed 47 h later by human chorionic gonadotropin (hCG, 4 IU intraperitoneally) (Pregynal, Organon, Oss, The Netherlands). They were then mated with males of the same strain overnight. Successful mating was confirmed by identification of a vaginal plug on the following morning (Day 1 of pregnancy).

On Day 4 of pregnancy (at 97 h post hCG administration), the fertilized mice were killed by cervical dislocation and their embryos isolated by flushing the uterus and oviducts using an in-house flushing-holding medium^59^ plus 1% bovine serum albumin (BSA). The collected embryos underwent immediate classification using a Nikon SMZ 745 T stereoscope (Nikon, Tokyo, Japan); only those at the blastocyst stage were selected for further analysis. Blastocysts from at least six blood-unrelated females were used in each fluorescence and immunochemical staining procedure (see below).

For experiments requiring positive controls*,* 50 blastocysts were treated with actinomycin D, an apoptotic inductor known to increase the frequency of apoptosis in mouse embryos (Supplementary Fig. S3, S4)^18^. Freshly isolated blastocysts were cultured under standard conditions (5% CO_2_, atmospheric O_2_ and 37 °C) for 24 h in synthetic oviduct medium (EmbryoMax KSOM [potassium simplex optimized medium] with amino acids and D-glucose [Millipore, Darmstadt, Germany]) supplemented with 0.1% embryo culture tested bovine serum albumin and 5 ng/ml actinomycin D.

### Analysis of phosphatidylserine-flipping in apoptotic embryonic cells

For the microscopic analysis of PtdSer-flipping in apoptotic embryonic cells, two staining kits were used: the Annexin-V-FLUOS Staining Kit (Roche Diagnostics, Penzberg, Germany), and the Annexin V-FITC Apoptosis Detection Kit (Sigma-Aldrich). Freshly isolated blastocysts were incubated in flushing-holding medium containing Hoechst 33342 cell-permeable DNA stain (20 µg/ml) for 15 min at 37 °C. They were then incubated in a mixture of binding buffer (an isotonic medium containing calcium, an element essential for the binding of AV to PtdSer), fluorescein isothiocyanate-conjugated AV (20 µg/ml) and PI (20 µg/ml) for 15 min at 37 °C (both reagents provided with the kits). Samples were then placed on pre-heated slides and examined at a magnification of × 400 by CLSM using a Leica TCS SPE microscope (Leica, Mannheim, Germany). The negative staining control omitted AV from the labelling protocol (Supplementary Fig. S3).

AV staining was performed to determine the presence of PtdSer residues on the outer surface of the plasma membrane (green labelling = AV +); PI staining of DNA was used to determine the integrity of the cell membrane (red labelling in nucleoplasm of dying cells = PI +). Hoechst 33342 DNA staining was performed to visualize nuclear morphology (provides blue labelling of the nucleoplasm of all cells) and to distinguish normal chromatin organization (oval nuclei or occasional mitotic configurations), chromatin condensation (pyknotic nuclei with significantly more intense labelling), and karyorrhexis (nuclei decayed into round fragments of condensed chromatin) (Fig. 1).

### Efferocytosis in mouse blastocysts

The microscopic analysis of apoptotic cell internalization in mouse blastocysts involved two methods of visualizing the plasma membrane: fluorescence staining of F-actin with phalloidin-TRITC conjugate (Santa Cruz Biotechnology, Inc., Santa Cruz, CA, USA), and the immunochemical staining of E-cadherin (red labelling in both cases, see detailed description below) (Fig. 2). For the microscopic analysis of phagosome formation, two assays were performed: the LysoTracker Kit was used to determine the formation of acid organelles (pinkish labelling, Fig. 3), and immunochemical staining was used to determine the presence of active form of RAC1 (green labelling, Fig. 4) (see detailed description below).

The same type of embryo fixation was performed during each staining protocol: freshly isolated blastocysts were fixed in 4% w/v paraformaldehyde (Merck, Darmstadt, Germany) in phosphate buffered saline (PBS) (Invitrogen Life Technologies) at room temperature for 10 min and stored in 1% w/v paraformaldehyde in PBS at 4 °C for up to one week as needed. All stained blastocysts were mounted on glass slides with Vectashield (Vector Laboratories, Burlingame, CA, USA) and examined by using a Leica TCS SPE confocal microscope (magnification × 400).

Each staining procedure (except the RAC1 immunochemistry) was combined with a TUNEL assay (terminal deoxynucleotidyl transferase dUTP nick end labelling) using a DeadEnd Fluorometric TUNEL System (Promega Corporation, Madison, USA) to track DNA fragmentation in the nucleoplasm of apoptotic cells (TUNEL +  = green), and with Hoechst 33342 DNA staining to visualize nuclear morphology and thus distinguish between normal chromatin organization, chromatin condensation and karyorrhexis (blue). Clusters of nuclear fragments taking up space comparable to the size of a normal nucleus were counted as one apoptotic nucleus. Nuclear fragments standing alone were considered remnants of apoptotic nuclei only when TUNEL + . As a positive staining control for each TUNEL assay, two to four randomly selected blastocysts were pre-incubated in 50 U /ml DNase I (Invitrogen Life Technologies, Karlsruhe, Germany) for 30 min at 37 °C, an enzyme inducing DNA nicks. A negative staining control was obtained by omitting the terminal transferase from the labelling procedure (Supplementary Fig. S4).

TUNEL + nuclei localized within embryonic cells with a normal nucleus and intact plasma membrane were classified as internalized apoptotic cells. TUNEL + nuclei surrounded with intact plasma membranes from at least three neighbouring cells were classified as non-internalized apoptotic cells. TUNEL + nuclei (or nuclei showing chromatin condensation) in the blastocoele cavity or perivitelline space were classified as extruded apoptotic cells.

#### F-actin fluorescence staining

Fixed blastocysts were washed in PBS containing 0.1% BSA and transferred into PBS with 0.5% v/v Triton X-100. After 1 h permeabilization, the blastocysts were washed in PBS with BSA and incubated in TUNEL assay reagents for 1 h at 37 °C in the dark^60^. After further washing in PBS with BSA, the embryos were transferred into a 3 µM solution of fluorescent phalloidin-TRITC (phalloidin tetramethylrhodamine B isothiocyanate) (Santa Cruz Biotechnology) prepared according to the manufacturer's instructions. Labelling took 30 min and was performed at room temperature. After final washing in PBS with BSA, the blastocysts were counterstained with Hoechst 33342 DNA (20 μl/ml in PBS with BSA) for 5 min at room temperature and mounted on glass slides. A negative staining control was obtained by omitting phalloidin-TRITC from the labelling protocol (Supplementary Fig. S5).

#### E-cadherin immunochemical staining

Fixed blastocysts were washed in PBS with BSA, permeabilized 1 h in 0.1% Triton X-100, incubated in TUNEL assay reagents for 1 h at 37 °C in the dark, and subjected to further washing in PBS with BSA^60^. Non-specific immunoreactions were blocked by incubating the embryos in a solution containing 10% v/v normal goat serum (Santa Cruz Biotechnology) and 2% w/v BSA fraction V in PBS for 2 h at room temperature. After blocking, the blastocysts were incubated with primary antibody (Mouse anti-E Cadherin antibody [M168]—C-terminal, 1:200 dilution) (Abcam, Cambridge, UK) diluted in blocking solution at 4 °C overnight. The next day, the blastocysts were extensively washed in the blocking solution and incubated with Alexa Fluor 594-conjugated AffiniPure Goat Anti-Mouse IgG secondary antibody (1:100 dilution) (Jackson ImmunoResearch Laboratories, West Grove, PA, USA) for 1 h at room temperature. Finally, the blastocysts were counterstained with Hoechst 33342 (20 μl/ml in PBS) for 5 min at room temperature and mounted on glass slides. Immunochemical negative control staining was performed by omission of either the primary or secondary antibody (Supplementary Fig. S5).

#### LysoTracker labelling

The LysoTracker Red DND-99 staining kit (Cell Signaling Technology, Inc., Danvers, MA, USA) was used to label acid organelles according to the manufacturer’s instructions. Briefly, freshly isolated blastocysts were washed in flushing-holding medium supplemented with BSA, and incubated in 50 nM LysoTracker Red DND-99 diluted in flushing-holding medium for 1 h at 37 °C. Subsequently, the blastocysts were fixed in 4% w/v paraformaldehyde (Merck) in PBS for 10 min at room temperature and stored in 1% w/v paraformaldehyde in PBS at 4 °C until the next day. Fixed blastocysts were washed in PBS with BSA, permeabilized for 1 h in 0.5% Triton X-100, incubated in TUNEL assay reagents for 1 h at 37 °C in the dark, and subjected to further washing in PBS with BSA^60^. Finally, the blastocysts were counterstained with Hoechst 33342 (20 μl/ml in PBS) for 5 min at room temperature and mounted on glass slides. A negative staining control was obtained by omitting LysoTracker Red DND-99 from the labelling protocol (Supplementary Fig. S5).

#### RAC1 immunochemical staining

The zona pellucida of freshly isolated blastocysts was removed with 0.5% w/v pronase in flushing-holding medium at 37 °C. Zona-free blastocysts were fixed in 4% w/v paraformaldehyde (Merck) in PBS for 10 min at room temperature, washed in PBS containing 0.1% BSA, and transferred into PBS with 0.5% v/v Triton X-100. After 1 h permeabilization, they were washed in PBS with BSA. Non-specific immunoreactions were blocked by incubating the embryos in 10% normal goat serum (Santa Cruz Biotechnology) at 4 °C overnight. After blocking, they were incubated with primary antibody (anti-active Rac1-GTP mouse monoclonal antibody, 1:50 dilution) (NewEast Biosciences, Malvern, PA, USA) diluted in blocking solution at 4 °C for 24 h. The next day, the blastocysts were extensively washed in blocking solution and incubated with goat anti-mouse secondary antibody conjugated with Fluorescein FITC (1:200) (Jackson ImmunoResearch Laboratories) diluted in blocking solution for 1 h at room temperature. Finally they were counterstained with Hoechst 33342 (20 μl/ml in PBS) for 5 min at room temperature and mounted on glass slides. A negative control staining was performed by omitting either the primary or secondary antibody (Fig. 4). TUNEL assay was excluded from staining protocol to avoid false-positive labelling.

#### Transmission electron microscopy

Six blastocysts were randomly selected for ultrastructural examinations of efferocytosis by TEM (Fig. 5). All embryos were fixed in 2.0% paraformaldehyde and 2.5% glutaraldehyde diluted in 0.1 M cacodylate buffer (pH 7.3) at 4 °C for 60 min. They were then washed in 0.1 M sodium cacodylate buffer twice for 15 min and post-fixed in 1% OsO4 in 0.1 M sodium cacodylate buffer for 1 h at room temperature. The blastocysts were then individually embedded in 2% agar. After dehydrating by passing them through an acetone series (30, 50, 70, 80, 90 and 100%), they were embedded in Poly/Bed resin (Polysciences Inc., Warrington, PA, USA) and serially sectioned (*c*80 nm) using an Ultracut UC 6 ultramicrotome (Leica, Wetzlar, Germany). These ultra-thin sections were collected on mesh nickel grids, contrasted with uranyl acetate and lead citrate, and examined using a JEOL CX100 transmission electron microscope (JEOL, Tokyo, Japan)^61^.

### Expression of selected receptors and immunoregulatory proteins involved in efferocytosis

Total RNA was extracted from batches of 80–120 mouse blastocysts, and from adult mouse liver, kidney, heart and brain (positive tissue controls). TRIzol Reagent (Invitrogen Life Technologies) was used for RNA extraction according to the manufacturer’s instructions. DNA contaminating the RNA preparations was digested with amplification grade DNase I (Invitrogen Life Technologies). The RNA (from 80–120 blastocysts or 0.5 μg of tissue RNA) was then reverse transcribed with Superscript III RNase H- Reverse Transcriptase (Invitrogen Life Technologies) using 4 μM anchored oligo dT primers and 1.5 μM random hexamer primers (Thermo Fisher Scientific ABgene, Epsom, UK). To check for the presence of genomic DNA contamination, reverse transcriptase negative controls (no reverse transcriptase in the reaction [‘RT minus’ reaction]) were performed in parallel using half of each RNA sample. The cDNA preparations were then cleaned by ethanol precipitation, and diluted in an appropriate amount of 10 mM Tris (pH 8.3) (1 μl of the cDNA corresponded theoretically to 2.5 blastocyst equivalents). Two independent cDNA preparations (prepared from separate embryo pools) were used in all analyses.

PCR amplifications were performed in 25 μl volumes containing 1 μl of cDNA (corresponding to 2.5 embryo equivalents or 0.5 μg tissue RNA), 0.5 μM of oligonucleotide primers (commercial primer sets from Qiagen [Valencia, CA]; Table 1), 50 mM KCl, 10 mM Tris–HCl pH 8.3, 2 mM MgCl_2_, 0.2 mM dNTPs, and 0.02 U/ml platinum Taq DNA polymerase (Invitrogen Life Technologies). An initial step at 94 °C for 2 min was followed by 40 cycles (blastocysts) or 35 cycles (positive tissue controls) at 94 °C for 30 s, 60 °C for 30 s, and 72 °C for 30 s. To check for the presence of cross contamination, a reaction with water instead of cDNA was performed concurrently (blank reaction). The detection of beta-actin transcripts, using beta-actin primers^62^, served as the control for RNA integrity and of the RT-PCR process. To evaluate the contribution of potential genomic DNA contamination to the PCR product, some amplifications were performed in a Light Cycler 480 real-time PCR system (Roche Diagnostics, Rotkreuz, Switzerland). The SYBR Green qPCR Mastermix (Qiagen), and the above-mentioned primers (Table 1) and thermal profile were employed, with an additional step at the end of each amplification cycle (72 °C for 20 s) for the acquisition of fluorescence. Amplification specificity was assessed by melting curve analysis. No PCR products were detected in most RT minus reactions. In six genes a weak specific PCR product (PCR product of the expected size) was detected in RT minus reactions, indicating weak genomic contamination in the RNA preparation (Supplementary Fig. S1). To verify the presence of such transcripts (for *Cd14, Cd44, Mbl2, Itgav, Itgb3, Scarf1*) in the blastocysts, Ct values (number of amplification cycles needed for reaching the threshold fluorescence^63,64^) for the RT + reactions (using the standard cDNA template) and corresponding RT- reactions (using an RT- ‘cDNA’ template) were compared. The Ct values in reactions with the standard cDNA template were usually over five cycles fewer than in reactions with RT minus ‘cDNA’ (Supplementary Table S2), indicating the expression of the examined transcripts in the blastocysts to have taken place.

PCR products were analysed using electrophoresis on 2% and 3% agarose gels stained with GelGreen (Biotium, Hayward, CA, USA). A 100 bp and a 20 bp DNA ladder (Jena Bioscience, Jena, Germany) were used as markers. PCR products were visualized with a Fusion FX7 imaging system (Vilber Lourmat, Collégien, France), and the size of DNA bands (PCR products) determined using Bio-1D analysis software version 15.03. (Vilber Lourmat; https://www.vilber.com).

### Statistical analysis

The results are expressed as arithmetical means ± standard deviation or percentages. To determine whether the data sets reflected a normal distribution, they were analysed using the D’Agostino-Pearson normality test. Differences between groups of blastocysts subjected to the different staining methods in terms of the number of dead cells, dead cells in the ICM and TE cell lineages, TUNEL + nuclei, and of internalized, non-internalized, extruded and non-classified TUNEL + nuclei were determined using the Mann–Whitney test or Kruskal–Wallis test (followed by Dunn's *post-hoc* test) as required. Differences between groups of blastocysts subjected to the different staining methods in terms of the proportion of blastocysts containing dead cells, proportion of dead cells localized in ICM and TE cell lineages, and proportion of internalized, non-internalized, extruded and non-classified TUNEL + nuclei were analysed using the Chi-squared test with one or three degrees of freedom as required. Significance was set at *P* < 0.05. All calculations were made using Prism software version 8.1.2. (GraphPad Software Inc., La Jolla, CA, USA; https://www.graphpad.com).

### Ethics approval

All experiments were performed in mice of the outbred CD-1 strain (Velaz, Prague, Czech Republic). Mice were maintained in plexiglass cages under standard conditions (temperature 22 ± 2 °C, humidity 55 ± 5%, 12:12 h light–dark cycle with lights on 6:00 a.m.), with free access to food and water. All animal experiments were approved by the Ethics Committee for Animal Experimentation of the Institute of Animal Physiology, and by the State Veterinary and Food Administration of the Slovak Republic, and were performed in accordance with Slovak legislation based on EU Directive 2010/63/EU on the protection of animals used for experimental and other scientific purposes. The study was carried out in compliance with the Animal Research: Reporting of In Vivo Experiments (ARRIVE) guidelines.

## Supplementary Information

Supplementary Information.

## Data Availability

The data that support the findings of this study are available from the corresponding author, Dušan Fabian, upon reasonable request.
