# Supplementary material for: Apoptotic cells in mouse blastocysts are eliminated by neighbouring blastomeres
Source: Sci Rep. 2021 Apr 29;11:9228. doi: 10.1038/s41598-021-88752-0 (PMC8085119; doi:10.1038/s41598-021-88752-0)
Supplement: Supplementary file 1 — Supplementary Information. [file 41598_2021_88752_MOESM1_ESM.pdf]

Supplementary information

Apoptotic cells in mouse blastocysts are eliminated by neighbouring blastomeres

Jozef Pisko, Alexandra Špirková, Štefan Čikoš, Lucia Olexiková, Veronika Kovaříková, Zuzana Šefčíková, Dušan Fabian

Supplementary Table S1. Receptors expressed by non-professional and professional phagocytes, and immunoregulatory proteins involved in efferocytosis.

Receptor	Cell type	Species	Known ligands	Reference
CD36 (SCARB3)	retinal pigment cells	human	PtdSer, oxid. lipids	50
STAB1 (CLEVER-1)	sinusoid. endothelial cells	mouse	PtdSer	52
SCARF1	endothelial cells	mouse	C1q-PtdSer	44
VnR (Integrin αVβ3)	fibroblasts	human	MFGE8-PtdSer, Vn	53
CD14	endothelial cells	human	phospholipids, LPS	8
ADGRB1 (BAI1)	macrophages	human, mouse	PtdSer	54
CD300LF	myelocytes	mouse	PtdSer	45
MSR1 (SR-AI)	macrophages	human	oxidized lipids, LPS	46
SCARB1 (SR-BI)	macrophages	human	oxidized lipids	7
CD44	macrophages	human	hyaluronan, fibrin	55
CD93 (C1QR1)	macrophages	human	moesin, C1q	48

Regulatory protein	Cell type	Species	Known ligands	Reference
SFTPA1	alveolar macrophages	rat	carbohydrates	49
MBL1, MBL2	macrophages	human	carbohydrates, CRT	48

PtdSer, phosphatidylserine; C1q, complement component 1q; MFGE8, lactadherin; Vn, vitronectin; VnR, vitronectin receptor; LPS, lipopolysaccharide; CRT, calreticulin

Supplementary Table S2 Difference between Ct values of RT+ and corresponding RT- reactions (ΔCt), and relative quantity of transcripts (expressed as fold change between RT+ and RT- reactions) when RT- reactions produced specific PCR products.

Gene	ΔCt	Fold change (RT+/RT-)
Cd14	-5.2	36.7
Mbl2	-4.4	21.1
Cd44	-3.6	12.1
Itgav	-10.4	1351.2
Itgb3	-9.2	588.1
Scarf1	-6.3	78.8

Relative quantities (fold changes between RT+ and RT- reactions) were calculated using the method of Čikoš and Koppel (2009)[64]. For Cd14 and Mbl2, both blastocyst samples weakly produced specific products in RT- controls (mean ΔCt values are shown). For Cd44, Itgav Itgb3 and Scarf1, only one blastocyst sample weakly produced specific products in RT-control reactions.

Supplementary Fig. S1

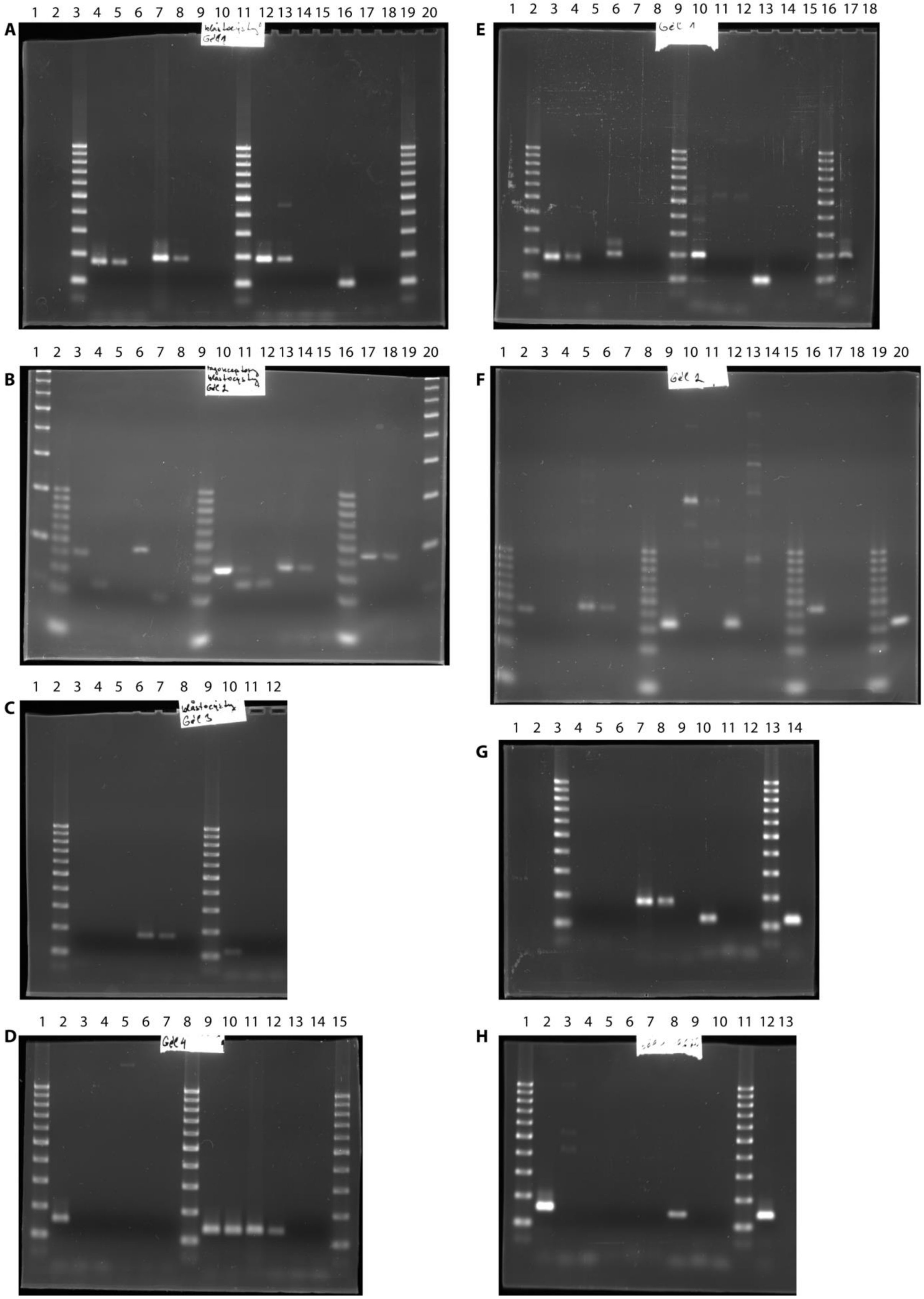

Supplementary Fig. S1 RT-PCR analysis of selected receptors and immunoregulatory proteins in mouse blastocysts. Two independent blastocyst samples were analysed. PCR products were analysed using electrophoresis in 2% and 3% agarose gels stained with GelGreen. A 100 bp DNA ladder (100 bp, 200 bp, 300 bp, 400 bp, 500 bp, 600 bp, 700 bp, 800 bp, 900 bp and 100 bp DNA fragments) and a 20 bp DNA ladder (20 bp, 40 bp, 60 bp, 80 bp, 100 bp, 120 bp, 140 bp, 160 bp, 180 bp and 200 bp DNA fragments) were used as markers. Agarose gels with separated PCR products are shown.

Gel A (Sample 1). Lanes: 3, 11 and 19, 100-bp DNA ladder; 4, *Cd14* RT+; 5, *Cd14* RT-; 6, *Cd14* blank; 8, *Cd36* RT+; 9, *Cd36* RT-; 10, *Cd36* blank; 13, *Cd93* RT+; 14, *Cd93* RT-; 15, *Cd93* blank; 16, *Cd300lf* RT+; 17, *Cd300lf* RT-; 18, *Cd300lf* blank.

Gel B (Sample 1). Lanes: 1 and 20, 100-bp DNA ladder; 2, 9 and 16, 20-bp DNA ladder; 3, *Adgrb1* RT+; 4, *Adgrb1* RT-; 5, *Adgrb1* blank; 6, *Cd44* RT+; 7, *Cd44* RT-; 8, *Cd44* blank; 10, *Itgav* RT+; 11, *Itgav* RT; 12, *Itgav* blank; 13, *Itgb3* RT+; 14, *Itgb3* RT-; 15, *Itgb3* blank; 17, *Scarf1* RT+; 18, *Scarf1* RT-; 19, *Scarf1* blank.

Gel C (Sample 1). Lanes: 2 and 9, 100-bp DNA ladder; 3, *Mbl1* RT+; 4, *Mbl1* RT-; 5, *Mbl1* blank; 6, *Mbl2* RT+; 7, *Mbl2* RT-; 8, *Mbl2* blank; 10, *Msr1* RT+; 11, *Msr1* RT-; 12, *Msr1* blank.

Gel D (Sample 1). Lanes: 1, 8 and 15, 100-bp DNA ladder; 2, *Scarb1* RT+; 3, *Scarb1* RT-; 4, *Scarb1* blank; 5, *Sftpa1* RT+; 6, *Sftpa1* RT-; 7, *Sftpa1* blank; 12, *Stab1* RT+; 13, *Stab1* RT-; 14, *Stab1* blank.

Gel E (Sample 2). Lanes: 2, 9 and 16, 100-bp DNA ladder; 3, *Cd14* RT+; 4, *Cd14* RT-; 5, *Cd14* blank; 6, *Cd36* RT+; 7, *Cd36* RT-; 8, *Cd36* blank; 10, *Cd93* RT+; 11, *Cd93* RT-; 12, *Cd93* blank; 13, *Cd300lf* RT+; 14, *Cd300lf* RT-; 15, *Cd300lf* blank.

Gel F (Sample 2). Lanes: 1, 8, 15 and 19, 20-bp DNA ladder; 2, *Adgrb1* RT+; 3, *Adgrb1* RT-; 4, *Adgrb1* blank; 5, *Cd44* RT+; 6, *Cd44* RT-; 7, *Cd44* blank; 9, *Itgav* RT+; 10, *Itgav* RT-; 11, *Itgav* blank; 12, *Itgb3* RT+; 13, *Itgb3* RT-; 14, *Itgb3* blank; 16, *Scarf1* RT+; 17, *Scarf1* RT-; 18, *Scarf1* blank.

Gel G (Sample 2). Lanes: 3 and 13, 100-bp DNA ladder; 4, *Mbl1* RT+; 5, *Mbl1* RT-; 6, *Mbl1* blank; 7, *Mbl2* RT+; 8, *Mbl2* RT-; 9, *Mbl2* blank; 10, *Msr1* RT+; 11, *Msr1* RT-; 12, *Msr1* blank.

Gel H (Sample 2). Lanes: 1 and 11, 100-bp DNA ladder; 2, *Scarb1* RT+; 3, *Scarb1* RT-; 4, *Scarb1* blank; 5, *Sftpa1* RT+; 6, *Sftpa1* RT-; 7, *Sftpa1* blank; 8, *Stab1* RT+; 9, *Stab1* RT-; 10, *Stab1* blank.

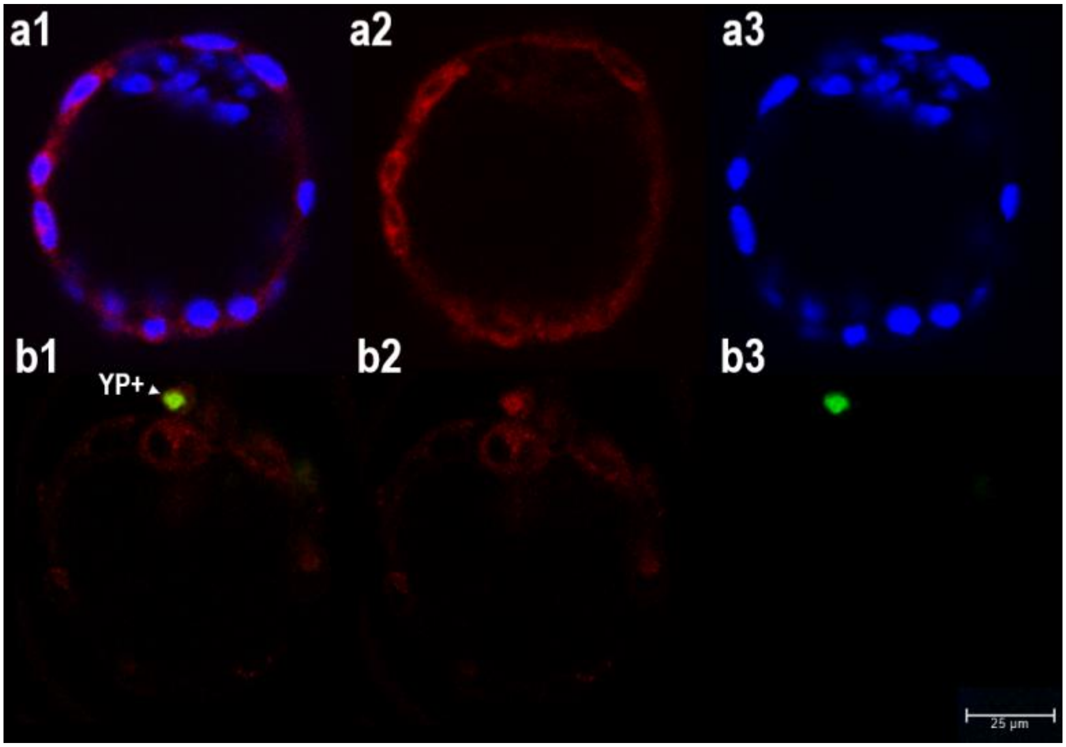

Supplementary Fig. S2 Analysis of apoptotic cell internalization in mouse blastocysts using alternative fluorescence staining. Images were obtained by confocal laser scanning microscopy (magnification x 400). Single optical sections of two blastocysts are shown in pictures a and b. The visualization of plasma membranes was attempted using lipophilic carbocyanine staining (Vybrant DiD, Molecular Probes Europe BV, Leiden, The Netherlands) (red). Apoptotic cells in living mouse blastocysts were identified via YO-PRO-1 staining (Molecular Probes, Inc., Eugene, OR, USA) (YP+, green). a: carbocyanine staining (a2, red) combined with Hoechst 33342 DNA staining (a3, blue) – membrane staining showed very diffuse signal (a1: merge). b: carbocyanine staining (b2, red) combined with YO-PRO-1 staining (b3, green) – after combination with apoptotic cell tracking the signal of membrane staining was significantly weakened (b1: merge). After fixing the embryos (necessary for the TUNEL assay) the signal of membrane staining was completely lost (data not shown).

Supplementary Fig. S3

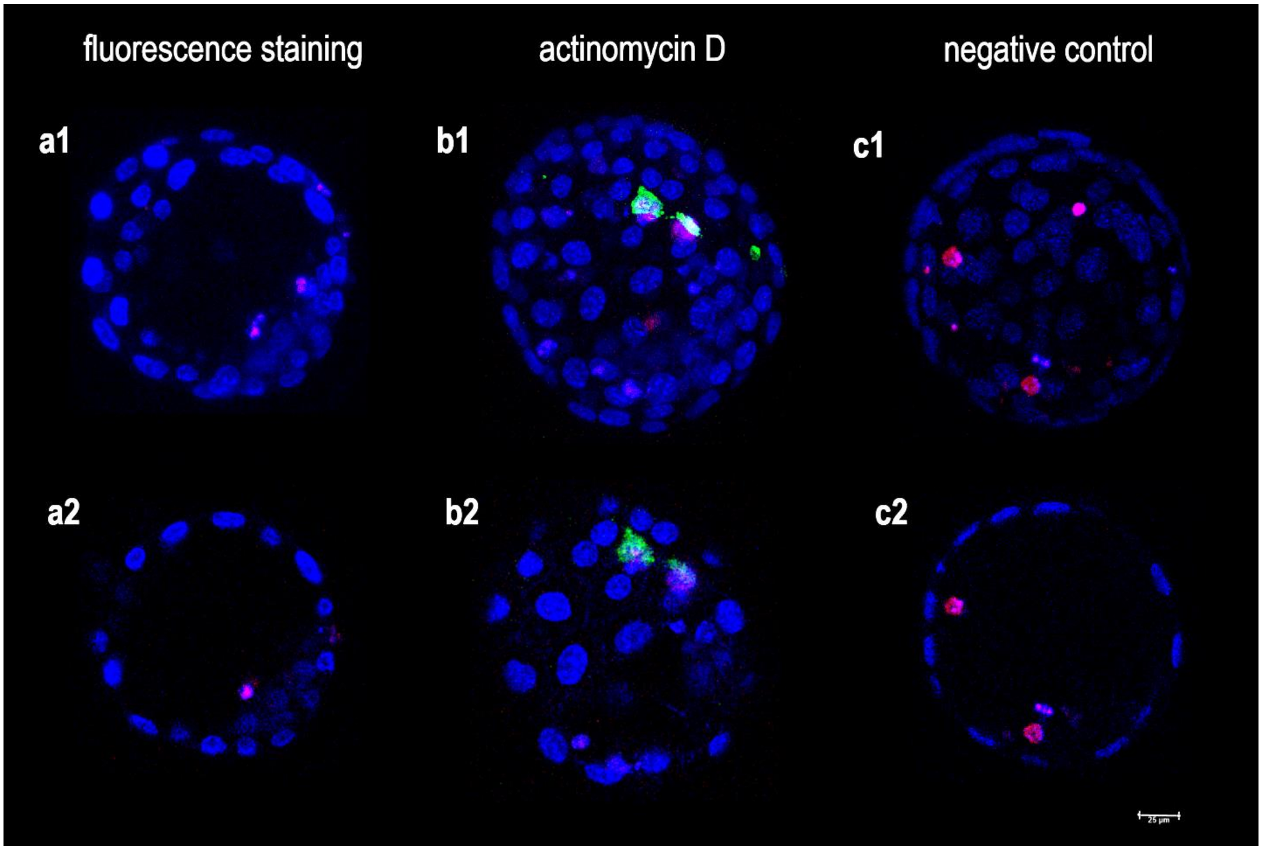

Supplementary Fig. S3 Positive and negative staining controls for the phosphatidylserine-flipping assay. Images were obtained by confocal laser scanning microscopy (magnification x 400). Overlays (1) and single optical sections (2) of three different blastocysts are shown in pictures a-c. Freshly recovered blastocysts were subjected to vital annexin V (green), propidium iodide (red) and Hoechst 33342 (blue) fluorescence staining (a). Positive control blastocysts were exposed to actinomycin D for 24 h *in vitro* (b). A negative staining control was obtained by omitting annexin V during the labelling of other anctinomycin D-treated blastocysts (c).

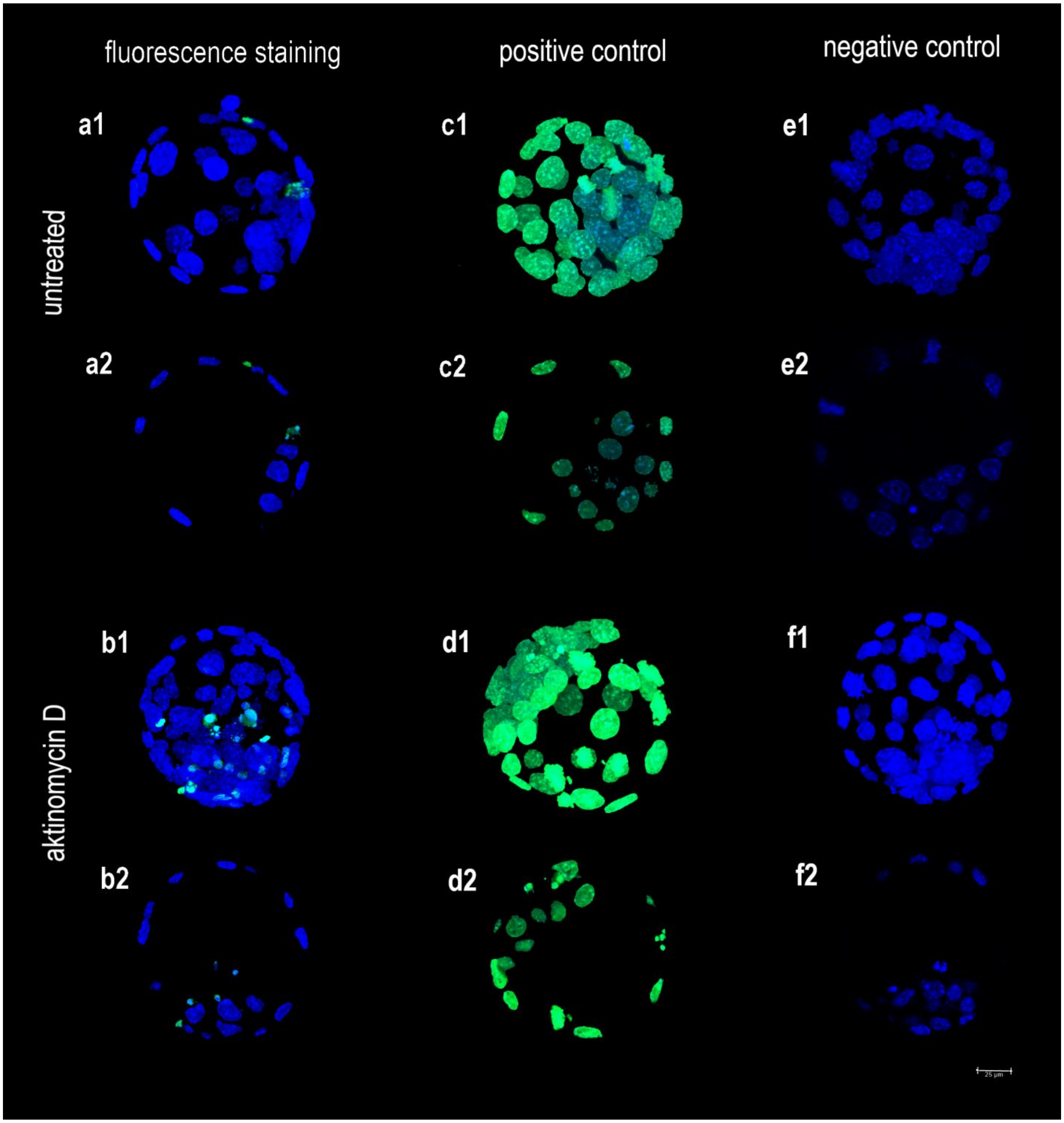

Supplementary Fig. S4 Positive and negative staining controls for DNA fragmentation assay. Images were obtained by confocal laser scanning microscopy (magnification x 400). Overlays (1) and single optical sections (2) of six different blastocysts are shown in pictures a-f. Freshly recovered blastocysts (a,c,e) and blastocysts exposed to actinomycin D for 24 h *in vitro* (b,d,f) were fixed and subjected to TUNEL labelling (green) and Hoechst 33342 (blue) fluorescence staining. A positive staining control was obtained by pre-incubation of fixed blastocysts in DNase I (c,d). A negative staining control was obtained by omitting terminal transferase from the labelling procedure (e,f).

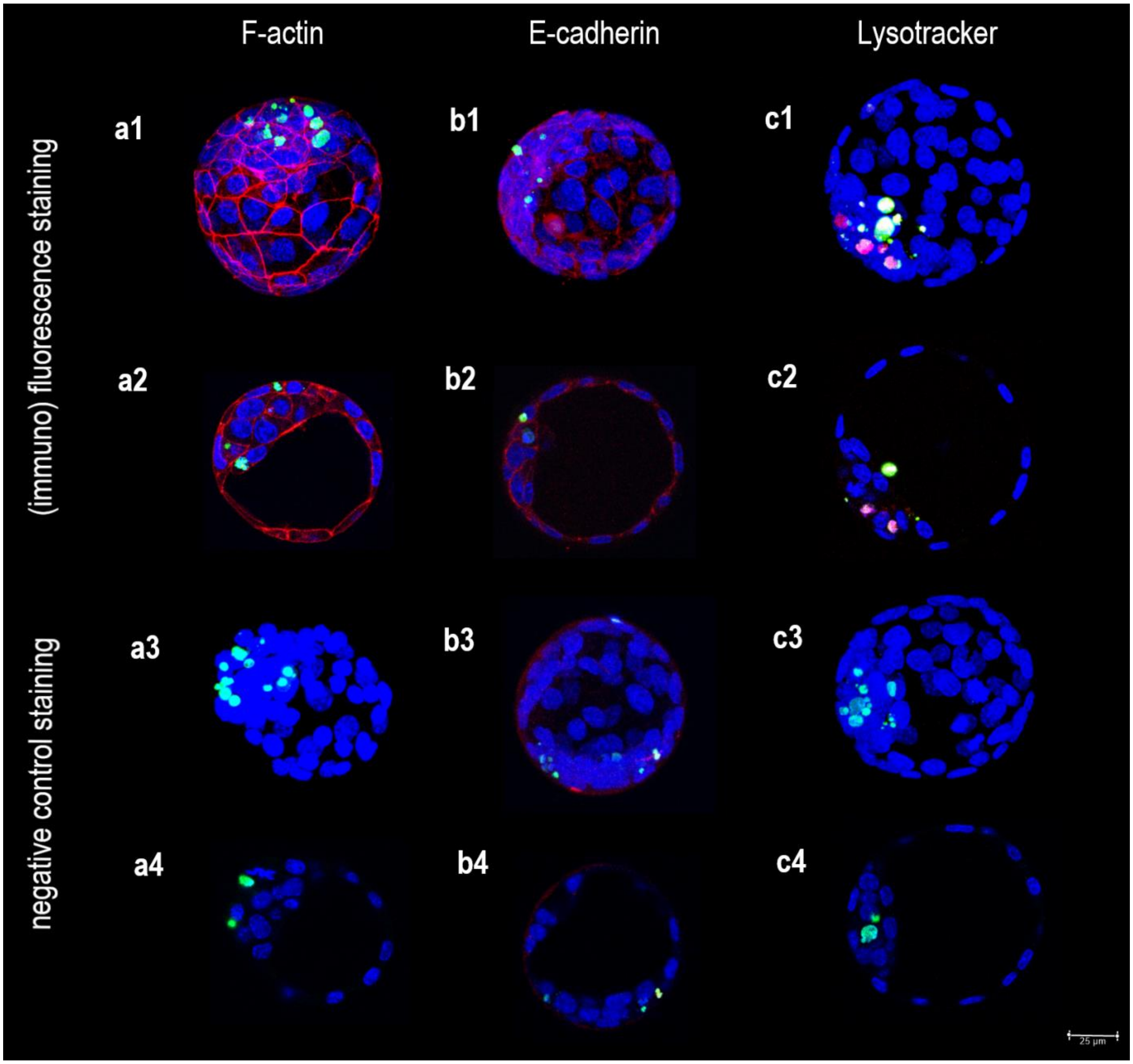

Supplementary Fig. S5 Negative staining controls for plasma membrane visualization and acid organelle tracking. Images were obtained by confocal laser scanning microscopy (magnification x 400). Overlays (1,3) and single optical sections (2,4) of six different blastocysts are shown. Recovered blastocysts were fixed and subjected to TUNEL labelling (green), Hoechst 33342 (blue) fluorescence staining, and fluorescence staining of F-actin with phalloidin-TRITC (a1-2, red) or immunochemical staining of E-cadherin (b1-2, red) or LysoTracker labelling (c1-2, red). Negative staining controls were obtained by omitting phalloidin-TRITC (a3-4), primary antibody (b3-4) or LysoTracker Red DND-99 (c3-4) from the labelling procedure.
